# Book Reviews

**Published:** 1799-05

**Authors:** 


### ANATOMY AND PHYSIOLOGY.

*Petri Camperi, ſummi medici, Diſſertationes decem, quibus ab illuſtribus Europæ, præcipue Galliæ academiis palma adjudicata.* Accedunt ejuſdem *de optima agendi vel expectandi in medicina ratione* liber ſingularis et diſſertatio *de forcipum indole et actione.* Cum tabulis in aere expreſſis. Vol. I. 1798. 8vo. pp. xiv. and 562. *Lingen.* Julicher.

The editor of this reſpectable collection is Mr. J. F. M. HERBELL, who is already known as a judicious and accurate compiler, by the edition he has publiſhed of the ſmaller tracts of the celebrated CAMPER, an author, whoſe great merit in medical ſcience, but particularly in ſurgery, anatomy, and phyſiology, are ſo univerſally acknowledged, that it is unneceſſary to enlarge upon them. We ſhall, therefore, content ourſelves with particularizing the ſubject of each treatiſe contained in the firſt volume: I. *De Infantum regimine,* written in 1762, and printed in *Tom.* VI. *Actor. Societatis Doctrinar. Hollandicæ.*—II. *De emolumentis et optima methodo inſitionis variolarum;* written in 1772, and firſt publiſhed at Groningen, in 1778, with two engravings, exhibiting the gradual changes of the puſtules after the inoculation of the ſmall-pox; a treatiſe which, at the preſent time, is peculiarly valuable.—III. *De incommodis ab unguentorum abuſu oriundis, et de eorum emendationibus in ulcerum curatione;* written in 1773, and printed in the fourth volume of the "*Memoires de l'Academie Royale de Chirurgie de Paris*", in 1779.—*De theoria et curatione morborum chronicorum pulmonum,* written in 1775, and never before printed. This treatiſe abounds in uſeful remarks and obſervations, relative to the morbid appearances noticed after diſſection.

diffection.—v. *Sur les Influentes,* &c. *i. e.* "On the influence which the air, by its different qualities, might difplay in chirurgical maladies; and on the means of treating them with fuccefs"; written in 1775, and now firft printed.—In this effay, the learned author endeavours to prove, that the conftitution of the atmofphere has no immediate influence on wounds and ulcers; that a great degree of heat or cold, alone, more fenfibly affect them; and that it is only from the general ftate or predifpofition of the body, that a wound will fuffer unfavourable changes through the infpiration of bad or corrupted air. vi. *De vera et præcipua caufa morborum, inter pecora et armenta epidemice, feu epizootice, graffantium;* written in 1777.

*Die trocknen Knochen, &c.*—"*A Defcription of the Bones of the human Body, in a dry State; for the ufe of his Pupils, and thofe who practife Anatomy at the Anatomical Theatre of Berlin:*" By Dr. J. G. Walter, &c. fourth Edition, improved. 8vo. 412 pp. with Plates. *Berlin.* Lange.

It muft be afcribed, in a great meafure, to the number of ftudents who frequent this inftitution at Berlin, and particularly all the natives who propofe to eftablifh themfelves in the Pruffian dominions, that this book has furvived a *fourth* edition. Although the defcriptions are, upon the whole, tolerably accurate, yet the ftyle of the author is uncommonly fatiguing, and his language frequently incorrect.

*Traité de Miologie,* &c.—"*A Treatife on Myology, according to the Method of Default;*" By Hyacinthe Gavard, his Pupil. 8vo. 398 pp. 1798. (4 livres) *Paris.* Méquignon, Sen. and the author.

This valuable treatife profeffedly owes its origin to the lectures of the late celebrated *Default,* which M. Gavard diligently attended and carefully tranfcribed.—The red or reddifh colour, however, fhould not have entered into the definition of a mufcle (pag. 1); for, in the human body, this is by no means a neceffary property of the mufcular fibre; and p. 17, the author again contradicts himfelf when he fays, that the red colour is not effentially prefent in the fibres of a mufcle. He diftinguifhes, with more propriety, between *fimple* and *compound* mufcles: in the former, the fibres lie only in one direction; while, in the latter, they interfect each other, and are varioufly interwoven one with another. With refpect to the tendons, page 23, we agree with him, that in the dead body they are more pliable than the mufcular fibres; but that the reverfe is the cafe in the living fubject. Among the properties inherent in mufcles, the author reckons elafticity, contractility—which he calls the '*dead power,*'— fenfibility, and irritability. The mufcular fibre may indeed be extended, but does not again contract with equal force; the contrary is obfervable in the tendinous fibre.—The mufcles poffefs but a fmall degree of fenfibility. *Harweg* adduces an inftance of necrofis, where the heart was laid bare: upon touching it, its motions became more violent, but the patient felt no pain. In treating of mufcular irritability, the author obferves, that the heart of a viper, after having feparated from it all the vifcera for fix hours, ftill manifefted the power of contraction. In diffecting various living animals, he never obferved a change of colour during the contraction of a mufcle.

In defcribing the individual mufcles, at confiderable length, the author has followed that method by which they can be difplayed to the greateft advantage, in anatomical preparations. Each mufcle he firft confiders according to its fuperior and inferior furface, and according to its fides, then he defcribes the tendon, and laftly, points out the effects of both.

Want of room does not permit us to ftate the peculiarities and deviations of of the author from other anatomiſts; we ſhall therefore only add, that in general he coincides in opinion with the celebrated *Sömmering*, and that this work, as may be expected from an ingenious pupil of Deſault, is replete with ſound and original remarks, on account of which it may juſtly be ranked among the claſſical books on the ſubject.

*Deſcription anatomique d'une tête humaine extraordinaire*, &c.—*An anatomical Deſcription of an extraordinary human Scull; with an Eſſay on the Origin of the Nerves:* By J. F. N. Jadelot. 8vo. with two Plates. (1 livre.) *Paris.*

The object of this deſcription, a human ſcull of an uncommon ſize, was found in the village of Sacy, near Rheims, about five feet from the ſurface of the ground, and is now preſerved in the valuable collection of Cit. Juſſieu. The author gives an account of its weight, dimenſions, and figure, compared with thoſe of the ordinary head of adults; and likewiſe furniſhes us with a chemical analyſis of its ſubſtance, compared with that of other human bones. In his opinion, this ſingular ſcull affords proof of a diſeaſe, before unknown, originating from a ſoftneſs of the bone (*molities oſſis*), accompanied with an enormous, but apparently ſyſtematic and regular ſwelling. He further conjectures, that the phoſphate of lime muſt have influenced the effect of the diſeaſe, ſo as to check its progreſs, while the carbonate of lime could not have increaſed till after the death of the ſubject.

